# Combined effects of water salinity and ammonia exposure on the antioxidative status, serum biochemistry, and immunity of Nile tilapia (*Oreochromis niloticus*)

**DOI:** 10.1007/s10695-023-01267-5

**Published:** 2023-11-21

**Authors:** Mahmoud A. O. Dawood, Mahmoud Gewaily, Hani Sewilam

**Affiliations:** 1 The Center for Applied Research On the Environment and Sustainability, The American University in Cairo, New Cairo, 11835 Cairo Egypt; 2 Animal Production Department, Faculty of Agriculture, Kafrelsheikh University, Kafr El-Sheikh, 33516 Egypt; 3 Department of Anatomy and Embryology, Faculty of Veterinary Medicine, Kafrelsheikh University, Kafr El-Sheikh, 33516 Egypt; 4 Department of Engineering Hydrology, RWTH Aachen University, Aachen, Germany

**Keywords:** Aquaculture, Brackish water, Cichlids, Sustainability, Water quality

## Abstract

Growing Nile tilapia in brackish water showed promising results, but the possibility of ammonia exposure can interrupt health status and productivity. Herein, the study tested the combined effects of water salinity and ammonia exposure on the antioxidative status, serum biochemistry, and immunity of Nile tilapia. Fish were assigned to eight groups where fish were reared in saline water (5, 10, and 15 ppt) with continuous or intermittent (every 3 days) total ammonia (TAN) exposure (5 mg TAN/L) (2 × 4 factorial design). After 30 days, the water salinity, TAN, and their interaction were markedly (*P* < 0.05), affecting the growth performance (final weight, weight gain, and specific growth rate) and survival rate of Nile tilapia. The growth performance and survival rate were markedly lower in tilapia grown in 15 ppt with continuous TAN exposure than in the remaining groups. The results showed that fish exposed to higher salinity levels (10 and 15 ppt) and continuous TAN exposure had a more robust antioxidative response, as evidenced by higher superoxide dismutase (SOD), catalase (CAT), and glutathione peroxidase (GPX) activities and lower malondialdehyde (MDA) levels in the homogenates of the gills, intestines, and livers. The gills were notably affected, with congestion of primary filaments blood vessels and degeneration or shedding of secondary filaments epithelium, especially at salinity levels of 10 and 15 ppt. Additionally, the intestines displayed hyperplasia and inflammatory cell infiltration of intestinal mucosa at 5–10 ppt salinity, degeneration and sloughing of the intestinal epithelium at 15 ppt saline water, and increased goblet cell number at salinity of 10 ppt. The study found that continuous TAN exposure had a more significant impact on the fish, especially at higher salinity levels. Water salinity, TAN, and their interaction significantly affected all measured blood bio-indicators (total, albumin, globulin, ALT, AST, creatinine, urea, glucose, and cortisol levels). The phagocytic activity and index were markedly lowered in fish reared in 15 ppt with continuous TAN exposure, while the lysozyme activity was decreased in fish grown in 5, 10, and 15 ppt with continuous TAN exposure. In conclusion, Nile tilapia showed the possibility of growth with normal health status in brackish water (5–10 ppt); however, continuous TAN exposure can impair the productivity of tilapia, especially with high salinity (15 ppt).

## Introduction

Nile tilapia (*Oreochromis niloticus*) has emerged as a promising aquatic animal due to its high yield and adaptability to various environmental conditions (El-Sayed 2019; FAO 2020). In addition, the potential of Nile tilapia for farming is further highlighted by its successful growth in brackish water conditions (de Godoy et al. 2022; Yu et al. 2022). The cultivation of Nile tilapia in brackish water with a salinity range of 10–12 ppt was accomplished without any detrimental effects on the growth performance, osmoregulation, immune response, and stress resistance of the fish (Garg et al. 2023; Ninh et al. 2014). Nile tilapia showed different responses when grown in different water salinity, which correlated with alterations in other factors, including temperature, level of dissolved oxygen, ammonia, and feed regimes (Dawood et al. 2021; Durigon et al. 2020). Indeed, salinity can affect the performances of fish through the modulation of osmoregulation, physiological, and metabolic functions (Marshall 2012; Mashaii et al. 2016).

Under intensive aquaculture conditions, tilapia can be stressed due to ammonia accumulation resulting from fish effluents, overfeeding, and organic nitrogen emissions (Manduca et al. 2021; Parvathy et al. 2023). High levels of accumulated ammonia cause multiple hazards to fish health, such as osmoregulation and physiological and metabolic disorders (Esam et al. 2022; Sánchez Ortiz et al. 2022). Consequently, fish suffer from oxidative stress, immunosuppression, and a high possibility of infection with diseases (Boyd 2017; Divya et al. 2020). Total ammonia nitrogen combines between the ionized (NH_4_^+^) and non-ionized (NH_3_^−^) ammonia forms and can be affected by the water temperature and pH value (Boyd and Tucker 2012). Un-ionized ammonia is the toxic form due to its capacity to diffuse through the membranes of gills (the hydrophobic micropores), leading to osmoregulation and oxidative stress features (Evans et al. 2006). In Nile tilapia, exposure to ammonia resulted in damage alterations in the gills that coincided with oxidative stress and immunosuppression (Abdo et al. 2022; Zhao et al. 2020). The ionization into NH_3_^−^ and NH_4_^+^ is greatly affected by the water’s salinity level. It has been reported that the decreasing salinity level at 20 °C can increase the ionization of NH_3_^−^ by 9% (Khoo et al. 1977; Kır et al. 2019; Sinha et al. 2015). Hence, the ammonia effects are more toxic in the case of low salinity levels, which has been confirmed in common carp (*Cyprinus carpio*) (Dawood and Sewilam 2023), European sea bass (*Dicentrarchus labrax*) (Kır et al. 2019), and Pacific white shrimp (*Litopenaeus vannamei*) (Long et al. 2021).

In Nile tilapia, the effects of different salinity levels on growth and health performances were investigated earlier (Caxico Vieira et al. 2018; Dawood et al. 2022b; Xu et al. 2018). Besides, ammonia exposure was tested in Nile tilapia (Benli et al. 2008; Esam et al. 2022). However, the combined impacts of ammonia and salinity levels on the performance and health status of Nile tilapia still need to be studied. Thus, the present study tested the continuous and intermittent effects of salinity and ammonia on the oxidative and immune responses as well as the histopathological alterations in the gills, liver, and spleen of Nile tilapia.

## Materials and methods

### Experimental procedure

Juveniles of Nile tilapia were brought from a private farm in the Kafrelsheikh area and received in two plastic tanks (1000 L) and kept for adaptation. The stock tanks were provided with continuous aeration while a third of the water was exchanged daily with free chlorine water. During the adaptation period, fish were offered a commercial diet with 30% crude protein (Aller Aqua Co., October City, Egypt) twice daily at 3% of the body weight. Then, the fish with a similar initial weight of 39.42 ± 1.2 g were distributed in 24 plastic tanks at 15 fish per tank. Each tank was enriched with continuous aeration and filled with 90 L water, where every three tanks represented one group. The eight groups were assigned to be grown under freshwater or saline water (5, 10, and 15 ppt) with continuous or intermittent (every 3 days) total ammonia (TAN) exposure (5 mg TAN/L) (2 × 4 factorial design). The division of the groups is presented in Table 1 where fish reared in G1: 0 ppt/continuous TAN; G2: 0 ppt/intermittent TAN; G3: 5 ppt/continuous TAN; G4: 5 ppt/intermittent TAN; G5: 10 ppt/continuous TAN; G6: 10 ppt/intermittent TAN; G7: 15 ppt/continuous TAN; G8: 15 ppt/intermittent TAN. The salinity level was raised gradually at 1 ppt daily using sea saline. Then after reaching the planned salinity levels, all fish were exposed to continuous or intermittent ammonia exposure at 5 mg TAN/L. The levels of TAN were decided based on the preliminary studies by Benli et al. (2008). To prepare TAN, an ammonium chloride (NH_4_Cl) (Sigma, St. Louis, MO, USA) solution was used to get the 5 mg TAN/L. A third of the water was replaced in all groups daily, and new chlorine-free water was added to keep the same saline levels with or without TAN. Fish were kept under these conditions for 30 days and fed on the same commercial diet at 3% of the body weight. The Portable Multiparameter Meter (Thermo Scientific™, Waltham, Massachusetts, USA) was used to detect the salinity, temperature, dissolved oxygen, and pH, while TAN levels were measured calorimetrically using the APHA (1912) standard method.

**Table 1:** Description of the experimental groups of Nile tilapia reared in water salinity and ammonia exposure

Group	Water salinity (ppt)	Ammonia exposure (5 mg TAN/L)	Description
G1	0	Every day	0 ppt/continuous ammonia
G2	0	Every 3 days	0 ppt/intermittent ammonia
G3	5	Every day	5 ppt/continuous ammonia
G4	5	Every 3 days	5 ppt/intermittent ammonia
G5	10	Every day	10 ppt/continuous ammonia
G6	10	Every 3 days	10 ppt/intermittent ammonia
G7	15	Every day	15 ppt/continuous ammonia
G8	15	Every 3 days	15 ppt/intermittent ammonia

### Final sampling

Then all fish were anesthetized with tricaine methane sulphonate (MS-222; 25 mg/L), and the blood was collected from 3 fish per tank from the caudal vein using 3 mL non-heparinized syringes. The collected blood was kept clotting at 4 °C, then serum was separated at 1107 g/15 min at 4 °C and kept at − 20 °C for further analysis. The intestines, livers, and gills were dissected from the fish to prepare the homogenate and stocked at − 20 °C. The homogenates of collected tissues were prepared by rinsing the tissues in ice-cold Phosphate-Buffered Saline (PBS) (50 mM potassium phosphate, pH 7.5 1 mM EDTA). Tissues were homogenized in tenfold PBS buffer (1 g tissue, 1:10 w:v) with glass homogenizer tubes (pellet pestle motor) and centrifuged at 7871 g for 5 min. The supernatant was collected and stored at 4 °C for further analysis.

### Biochemical analysis

Serum aspartate aminotransferase (AST), alanine aminotransferase (ALT), creatinine, and urea were detected by SPIN 800 Autoanalyzer using readymade chemicals (kits) supplied by Spinreact Co. Spain, following the manufacturer’s instructions. Serum total proteins and albumins were determined, according to Doumas et al. (1981) and Dumas and Biggs (1972). Globulin was calculated by the difference between serum total protein and albumins. Glucose and cortisol levels (MG/100 ml) were determined using glucose enzymatic PAP kits obtained from Bio-Merieux (France) (Trinder 1969).

Superoxide dismutase (SOD), catalase (CAT), and glutathione peroxidase (GPX) in gills, intestines, and livers homogenate samples were measured using commercial kits following the manufacturer’s (Biodiagnostics Co., Egypt) instructions. The concentration of malondialdehyde (MDA) was detected by following Uchiyama and Mihara (1978) and expressed as nmol MDA/g.

Serum lysozyme activity was determined using turbidimetric assay, according to the method described by Ellis (1990). One unit of lysozyme was defined as a reduction in absorbance of 0.001/min.

Phagocytic activity was determined according to Kawahara et al. (1991). To calculate the phagocytic index according to the following equations, the numbers of phagocytized and phagocytic cells were counted: phagocytic activity = macrophages containing yeast/total number of macrophages × 100; phagocytic index = number of cells phagocytized/number of phagocytic cells.

### Histomorphology sections

Gills, liver, and intestine were removed and flushed with PBS (pH 7.4) and fixed in neutral buffered formaldehyde for 48 h. The fixed specimens were processed by the conventional paraffin embedding technique, including the dehydration through ascending grades of ethanol, clearing in three changes of xylene, and melted paraffin ended by embedding in paraffin wax at 65 °C. Four µm thick sections were stained by Hematoxylin and Eosin (H and E), as previously described by Bancroft and Layton (2013). The tissue histopathology examination was carried out using a digital camera (Leica EC3, Leica, Germany) connected to a microscope (Leica DM500) and with software (Leica LAS EZ).

### Calculation and statistical analysis

After 4 weeks, all fish were starved for 24 h and then weighed and counted to calculate the growth performance, feed conversion ratio, and survival rate using the following formulae:

$$\begin{array}{l}\mathrm{Weight}\;\mathrm{gain}(\%)\hspace{0.17em}=\hspace{0.17em}((\mathrm{final}\;\mathrm{weight}\left(\mathrm g\right)-\mathrm{initial}\;\mathrm{weight}(\mathrm g))/\mathrm{initial}\;\mathrm{weight}(\mathrm g))\hspace{0.17em}\times\hspace{0.17em}100\\\mathrm{Specific}\;\mathrm{growth}\;\mathrm{rate}(\mathrm{SGR})\hspace{0.17em}=\hspace{0.17em}100\hspace{0.17em}\times\hspace{0.17em}\lbrack\mathrm{lnfinal}\;\mathrm{weight}\left(\mathrm g\right)-\ln\;\mathrm{initial}\;\mathrm{weight}(\mathrm g)\rbrack/\mathrm{days}\\\begin{array}{l}\mathrm{Feed}\;\mathrm{conversion}\;\mathrm{ratio}(\mathrm{FCR})\hspace{0.17em}=\hspace{0.17em}\mathrm{feed}\;\mathrm{intake}(\mathrm g)/((\mathrm{final}\;\mathrm{weight}\left(\mathrm g\right)-\mathrm{initial}\;\mathrm{weight}(\mathrm g))\\\mathrm{Survival}(\%)\hspace{0.17em}=\hspace{0.17em}100\hspace{0.17em}\times\hspace{0.17em}\mathrm{final}\;\mathrm{number}/\mathrm{initial}\;\mathrm{number}\;\mathrm{of}\;\mathrm{fish}\end{array}\end{array}$$

All data were analyzed using one-way analysis of variance (ANOVA) by the SPSS 22.0 software. Differences were considered significant at *P* < 0.05. When significant differences were detected, two-way ANOVA was used to determine the effects of water salinity, ammonia exposure, and their interaction on the performances of Nile tilapia.

## Results

### Water quality

The water quality indices in the tanks used for growing Nile tilapia in different salinity levels with continuous or intermittent ammonia exposure are shown in Table 2. The dissolved oxygen level ranged between 6.07 ± 0.19 and 6.31 ± 0.24 mg/L without significant differences among the groups. Further, the temperature (26.11 ± 0.49 to 26.51 ± 0.72 °C) and pH (6.19 ± 0.19 to 6.57 ± 0.34) did not show any different significances among the groups throughout the trial. The salinity levels showed marked differences among the groups where tilapia are grown in 0 ppt (G1 and G2) (0.26 ± 0.03 to 0.24 ± 0.02‰) had lower salinity levels than those reared in 5 ppt (G3 and G4) (5.09 ± 1.2 to 5.11 ± 0.24‰), 10 ppt (G5 and G6) (10.19 ± 0.29 to 10.21 ± 0.36‰), or 15 ppt (G7 and G8) (15.17 ± 0.41 to 15.23 ± 0.36‰) (*P* < 0.05). The continuous total ammonia (TAN) (5.03 ± 0.24 to 5.17 ± 0.28 mg/L) and un-ionized ammonia (NH_3_^−^) (0.068 ± 0.001 to 0.078 ± 0.001 mg/L) showed no marked changes among the groups. Similarly, the TAN (0.15 ± 0.01 to 0.27 ± 0.03 mg/L) and NH_3_^−^ (0.002 ± 0.0001 to 0.004 ± 0.0001 mg/L) showed insignificant differences when the exposure did not apply in tilapia tanks.

**Table 2:** Water quality for Nile tilapia reared in water salinity and ammonia exposure

Item	G1	G2	G3	G4	G5	G6	G7	G8	Two-way ANOVA (*P*-value)		
									Salinity	Ammonia	Interaction
Dissolved oxygen (mg/L)	6.27 ± 0.21	6.19 ± 0.12	6.07 ± 0.19	6.08 ± 0.16	6.17 ± 0.32	6.31 ± 0.24	6.28 ± 0.29	6.19 ± 0.23	NS	NS	NS
Salinity (‰)	0.24 ± 0.02d	0.26 ± 0.03d	5.09 ± 1.2c	5.11 ± 0.24c	10.21 ± 0.36b	10.19 ± 0.29b	15.23 ± 0.36a	15.17 ± 0.41a	0.001	NS	NS
Temperature (°C)	26.29 ± 0.37	26.35 ± 0.43	26.48 ± 0.52	26.37 ± 0.62	26.51 ± 0.72	26.11 ± 0.49	26.19 ± 0.52	26.48 ± 0.37	NS	NS	NS
pH	6.28 ± 0.16	6.57 ± 0.21	6.49 ± 0.29	6.19 ± 0.19	6.28 ± 0.31	6.38 ± 0.24	6.42 ± 0.27	6.57 ± 0.34	NS	NS	NS
Intermittent TAN (mg/L)	5.03 ± 0.24	5.06 ± 0.18	5.11 ± 0.16	5.09 ± 0.31	5.17 ± 0.28	5.16 ± 0.19	5.04 ± 0.47	5.08 ± 0.33	NS	NS	NS
Intermittent NH_3_-N (mg/L)	0.068 ± 0.001	0.076 ± 0.002	0.077 ± 0.002	0.076 ± 0.001	0.078 ± 0.001	0.077 ± 0.001	0.076 ± 0.002	0.076 ± 0.001	NS	NS	NS
Without TAN (mg/L)	0.23 ± 0.01	0.22 ± 0.02	0.26 ± 0.01	0.27 ± 0.03	0.18 ± 0.02	0.15 ± 0.01	0.26 ± 0.04	0.23 ± 0.02	NS	NS	NS
Without NH_3_-N (mg/L)	0.0022 ± 0.0001	0.003 ± 0.0001	0.004 ± 0.0001	0.0041 ± 0.0001	0.003 ± 0.0001	0.002 ± 0.0001	0.004 ± 0.0001	0.003 ± 0.0001	NS	NS	NS

Means ± S.E. in the same column with different letters differs significantly (*P* < 0.05). G1: 0 ppt/continuous total ammonia nitrogen exposure (TAN); *NS*, not significant (*P* ˃ 0.05); G2: 0 ppt/intermittent TAN; G3: 5 ppt/continuous TAN; G4: 5 ppt/intermittent TAN; G5: 10 ppt/continuous TAN; G6: 10 ppt/intermittent TAN; G7: 15 ppt/continuous TAN; G8: 15 ppt/intermittent TAN

### Growth performance

The water salinity, total ammonia exposure (TAN), and their interaction were markedly (*P* < 0.05) affecting the growth performance (final weight, weight gain, and specific growth rate) and survival rate of Nile tilapia (Table 3). Nile tilapia in G1, which reared in 5 ppt and intermittent exposure of TAN, showed higher growth performance, while fish in G7 (15 ppt/continuous TAN), which grew in 15 ppt and continuous TAN exposure, had lower growth indices than the remaining groups. Tilapia in G3 (5 ppt/continuous TAN), G5 (10 ppt/continuous TAN), G6 (10 ppt/intermittent TAN), and G8 (15 ppt/intermittent TAN) had similar growth performances with lower values than in G4 (5 ppt/intermittent TAN). The survival rate was markedly lower in G7 than in the remaining groups. Further, tilapia in G8 had a lower survival rate (86.67 ± 4.41%) than G1–G6 and higher than G7 (81.67 ± 4.41%). The remaining groups (G1–G6) did not show marked differences in the survival rate (95.00 ± 2.89 to 100.00 ± 0.00%).

**Table 3:** Growth performance of Nile tilapia reared in water salinity and ammonia exposure

Item	G1	G2	G3	G4	G5	G6	G7	G8	Two-way ANOVA (*P*-value)		
									Salinity	Ammonia	Interaction
IW (g)	39.45 ± 0.08	39.32 ± 0.12	39.53 ± 0.15	39.30 ± 0.23	39.23 ± 0.26	39.65 ± 0.06	39.52 ± 0.15	39.37 ± 0.13	NS	NS	NS
FW (g)	71.17 ± 2.69b	81.07 ± 0.99a	65.46 ± 2.55c	72.55 ± 2.21b	62.72 ± 0.57c	63.38 ± 1.41c	57.24 ± 1.93d	64.01 ± 3.00c	0.001	0.001	0.001
WG (%)	80.41 ± 6.69b	106.20 ± 2.86a	65.54 ± 5.97c	84.62 ± 5.77b	59.86 ± 0.58c	59.87 ± 3.77c	44.88 ± 5.38d	62.59 ± 7.59c	0.001	0.001	0.001
SGR (%/day)	0.98 ± 0.06b	1.21 ± 0.02a	0.84 ± 0.06c	1.02 ± 0.05b	0.78 ± 0.01c	0.78 ± 0.04c	0.62 ± 0.06d	0.81 ± 0.08c	0.001	0.001	0.001
Survival (%)	95.00 ± 2.89a	100.00 ± 0.00a	98.33 ± 1.67a	100.00 ± 0.00a	95.00 ± 2.89a	100.00 ± 0.00a	81.67 ± 4.41c	86.67 ± 4.41b	0.001	0.001	0.001

Means ± S.E. in the same column with different letters differs significantly (*P* < 0.05). *IW*, initial weight; *FW*, final weight; *WG*, weight gain; *SGR*, specific growth rate; *NS*, not significant (*P* ˃ 0.05); G1: 0 ppt/continuous total ammonia nitrogen exposure (TAN); G2: 0 ppt/intermittent TAN; G3: 5 ppt/continuous TAN; G4: 5 ppt/intermittent TAN; G5: 10 ppt/continuous TAN; G6: 10 ppt/intermittent TAN; G7: 15 ppt/continuous TAN; G8: 15 ppt/intermittent TAN

### Antioxidative responses

#### Gills

**Fig. 1:**
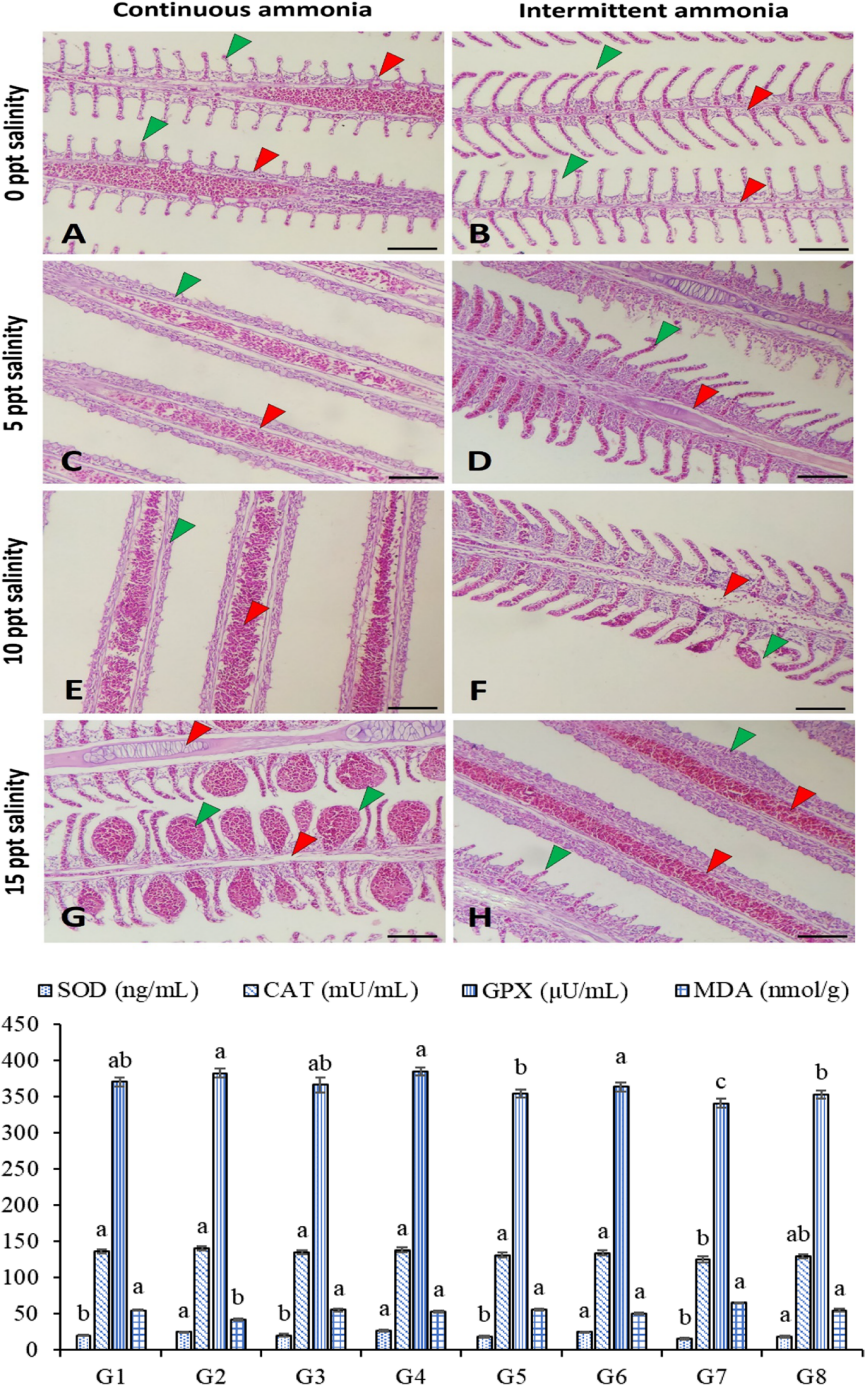
Photomicrograph of Nile tilapia gills subjected to continuous (left panel) and intermittent (right panel) ammonia with ascending levels of salinity (0 ppt; **A**–**B**, 5 ppt; **C**–**D**, 10 ppt; **E**–**F**, and 15 ppt; G–H) showed congestion of primary filaments (red arrowhead) blood vessels, degeneration or shedding of secondary filaments (green arrowhead) epithelium, and increased number of chloride cells particularly during continuous ammonia exposure, in addition, to severe telangiectasia at 15 ppt. Stain H&E. Bar = 100 µm. The antioxidative capacity of the liver homogenate is presented in the bars with eight groups. G1: 0 ppt/continuous total ammonia nitrogen exposure (TAN); G2: 0 ppt/intermittent TAN; G3: 5 ppt/continuous TAN; G4: 5 ppt/intermittent TAN; G5: 10 ppt/continuous TAN; G6: 10 ppt/intermittent TAN; G7: 15 ppt/continuous TAN; G8: 15 ppt/intermittent TAN

The gills’ antioxidative response to the different salinity levels and TAN exposure is presented in Fig. 1. The superoxide dismutase (SOD) was markedly higher in G2, G4, G6, and G8 than in G1, G3, G5, and G7. In addition, the catalase activity (CAT) was higher in G1–G6 than in G7 without significant differences with G8. The glutathione peroxidase (GPX) in G2, G4, and G6 is higher than in G5, G7, and G8 without significant differences with G1 and G3. However, GPX in G7 is lower than in G8. The malondialdehyde level (MDA) was significantly increased in all groups except for G2, which reared in 5 ppt with intermittent TAN exposure.

#### Intestines

**Fig. 2:**
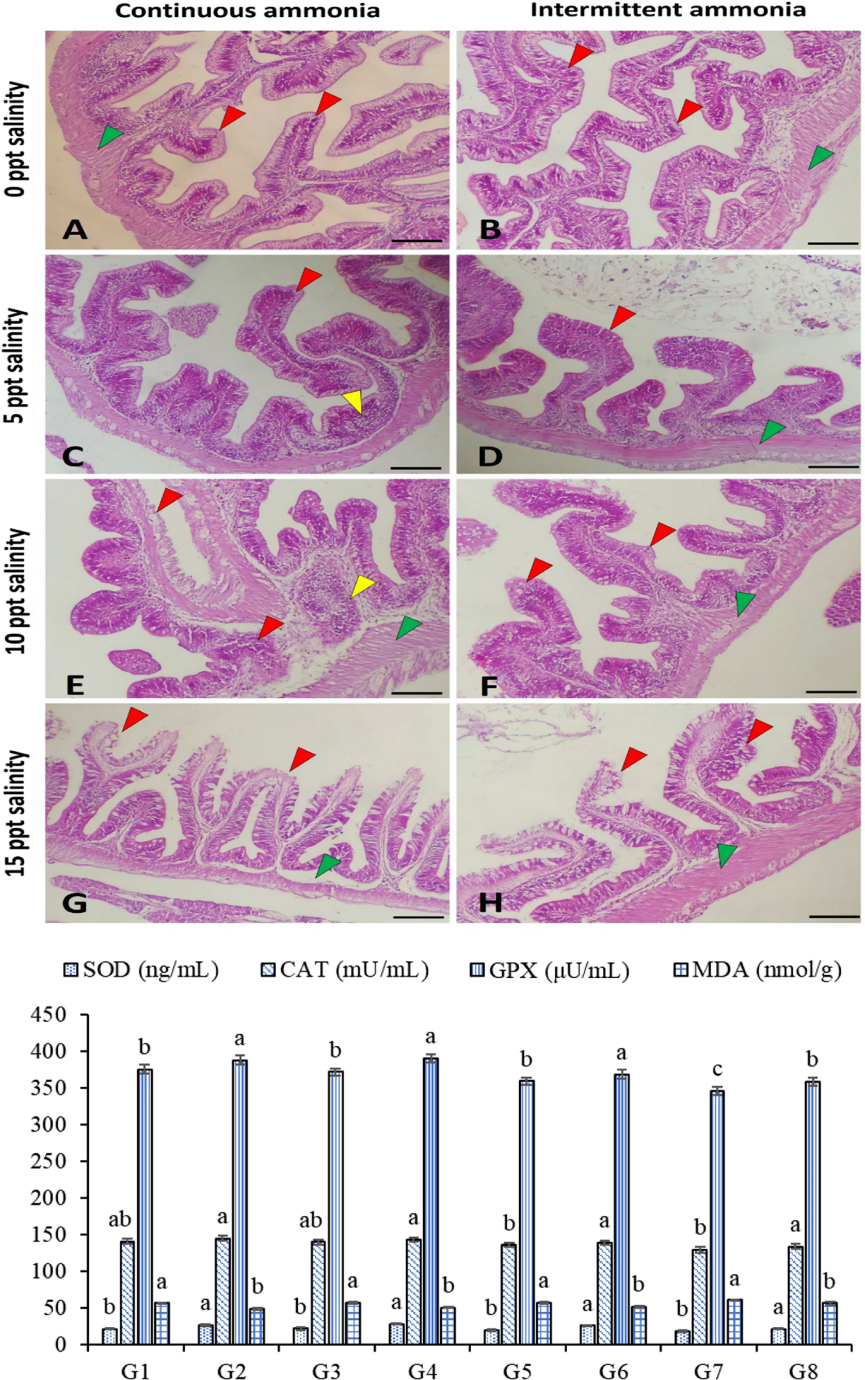
Photomicrograph of Nile tilapia intestine subjected to continuous (left panel) and intermittent (right panel) ammonia with ascending levels of salinity (0 ppt; **A**–**B**, 5 ppt; **C**–**D**, 10 ppt; **E**–**F**, and 15 ppt; **G**–**H**) showed apparently normal intestinal villi at 0 ppt, intestinal wall hyperplasia (green arrowhead) and inflammatory cell infiltration (yellow arrowhead) at 5–10 ppt, and degeneration and sloughing of the intestinal epithelium (red arrowhead) at 15 ppt salinity. Stain H&E. Bar = 100 µm. The antioxidative capacity of the liver homogenate is presented in the bars with eight groups. G1: 0 ppt/continuous total ammonia nitrogen exposure (TAN); G2: 0 ppt/intermittent TAN; G3: 5 ppt/continuous TAN; G4: 5 ppt/intermittent TAN; G5: 10 ppt/continuous TAN; G6: 10 ppt/intermittent TAN; G7: 15 ppt/continuous TAN; G8: 15 ppt/intermittent TAN

The intestines’ antioxidative response to the different salinity levels and TAN exposure is presented in Fig. 2. The SOD was markedly higher in G2, G4, G6, and G8 than in G1, G3, G5, and G7. In addition, the CAT was higher in G2, G4, G6, and G5 than in G7 without significant differences with G1 and G3. The GPX in G2, G4, and G6 is higher than in G1, G3, G5, G7, and G8. Fish in G7 had the lowest GPX significantly among all groups. However, GPX in G7 is lower than in G8. The MDA level was markedly lower in G2, G4, G6, and G8 than in G1, G3, G5, and G7.

#### Livers

**Fig. 3:**
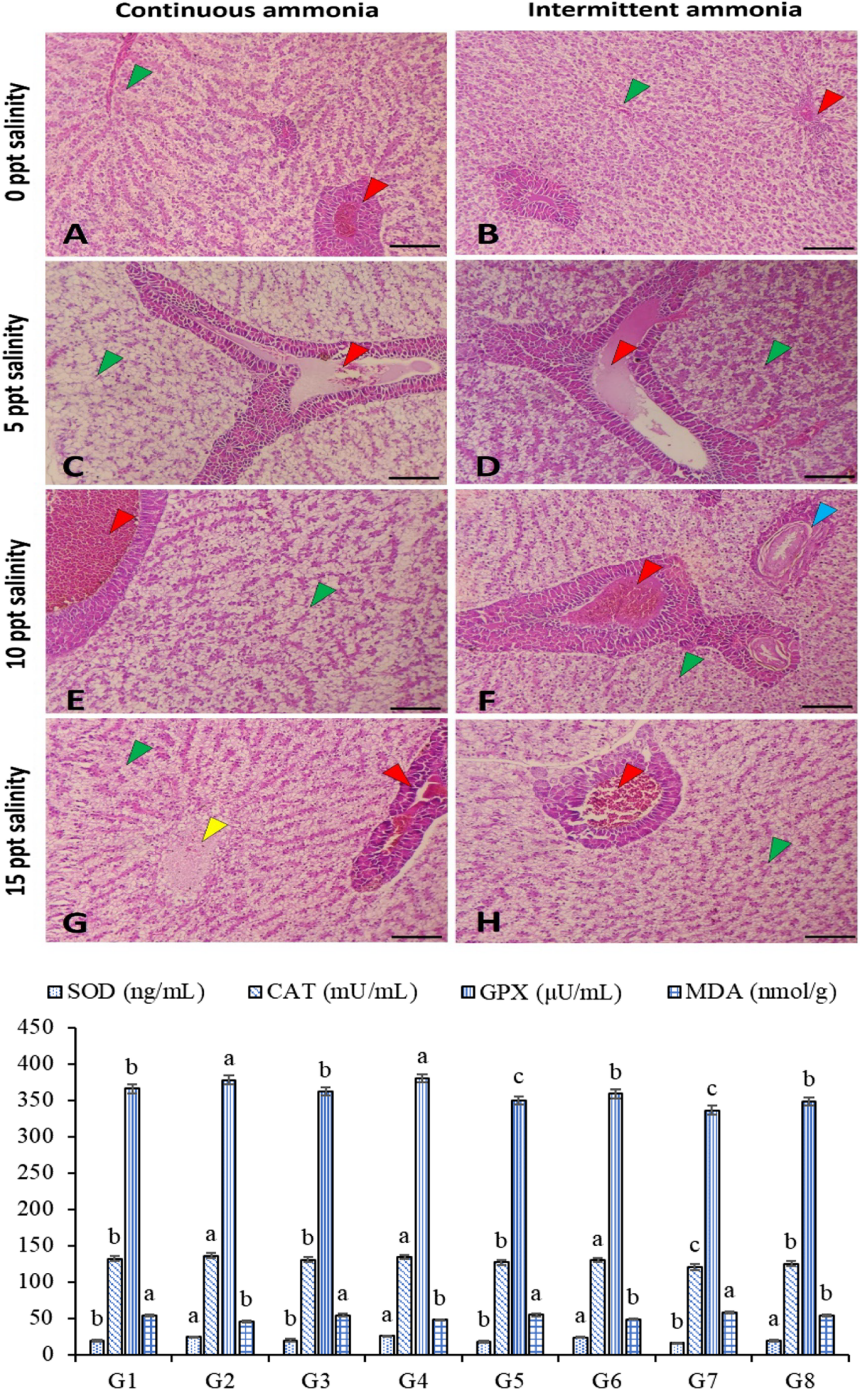
Photomicrograph of Nile tilapia liver subjected to continuous (left panel) and intermittent (right panel) ammonia with ascending levels of salinity (0 ppt; **A**–**B**, 5 ppt; **C**–**D**, 10 ppt; **E**–**F**, and 15ppt; **G**–**H**) showed slight vascular congestion in the freshwater groups at 0 ppt salinity (**A**, **B**), vascular dilation and edema at 5 ppt (**C**, **D**), severe vascular congestion of central veins and blood sinusoids at 10 ppt (**E**, **F**). At a higher level of salinity (15 ppt) with ammonia, there was vacuolation and necrosis of hepatocytes (**G**, yellow arrowhead) in addition to vascular congestion (**G**,** H**, green arrowhead) and perivascular pancreatic cell degeneration (**G**, **H**, red arrowhead). Stain H&E. Bar = 100 µm. The antioxidative capacity of the liver homogenate is presented in the bars with eight groups. G1: 0 ppt/continuous total ammonia nitrogen exposure (TAN); G2: 0 ppt/intermittent TAN; G3: 5 ppt/continuous TAN; G4: 5 ppt/intermittent TAN; G5: 10 ppt/continuous TAN; G6: 10 ppt/intermittent TAN; G7: 15 ppt/continuous TAN; G8: 15 ppt/intermittent TAN

The livers’ antioxidative response to the different salinity levels and TAN exposure is presented in Fig. 3. The SOD was markedly higher in G2, G4, G6, and G8 than in G1, G3, G5, and G7. In addition, the CAT was higher in G2, G4, and G6, and G than in G1, G3, G5, G7, and G8. Besides, G7 had a lower CAT than G8. The GPX in G2 and G4 is higher than in the remaining groups, while the GPX in G7 is lower than in the remaining groups. Fish in G7 had the lowest GPX significantly among all groups. However, GPX in G7 is lower than in G8. The MDA level was markedly lower in G2, G4, G6, and G8 than in G1, G3, G5, and G7.

### Histological study

The histopathological investigation of Nile tilapia gills, intestine, and liver was affected by increased salinity combined with TAN exposure. The hazard effect was generally more robust with continuous TAN exposure with increased salinity levels.

#### Gills

The gills of Nile tilapia were the most affected investigated organ (Fig. 1A–H). The histopathological findings include congestion of primary filaments blood vessels, degeneration or shedding of secondary filaments epithelium, and increased number of chloride cells, particularly at salinity > 5 ppt. Moreover, the gills tissue of fish subjected to both continuous TAN with high salinity dose suffered from severe congestion and telangiectasia of the secondary epithelium (Fig. 1G) or even complete shedding of the epithelial lining during intermittent TAN exposure (Fig. 1H).

#### Intestines

The intestines of all experimental fish appeared intact (Fig. 2A, B). The intestinal wall hyperplasia and inflammatory cell infiltration of intestinal mucosa were found at 5–10 ppt salinity (Fig. 2C–F). Furthermore, degeneration and sloughing of the intestinal epithelium were prominent at 15 ppt saline water (Fig. 2G, H) and increased goblet cell number at a salinity of 10 ppt. There was no significant difference between continuous and intermittent exposure to TAN.

#### Livers

The histopathological effect of salinity with TAN on the liver of Nile tilapia appeared in the form of slight vascular congestion at 0 ppt salinity (Fig. 3A, B), vascular dilation, and edema at 5 ppt (Fig. 3C, D), severe vascular congestion of central veins and blood sinusoids at 10 ppt (Fig. 3E, F). While at a higher level of salinity (15 ppt) with TAN, there was vacuolation and necrosis of hepatocytes in addition to vascular congestion and perivascular pancreatic cell degeneration. (Fig. 3G, H).

### Blood biomarkers

All measured blood bio-indicators were significantly affected by water salinity, TAN, and their interaction (Table 4). The blood proteins (total, albumin, and globulin) were markedly higher in G2 and G4 than in the remaining groups. At the same time, G7 and G8 had lower blood proteins than the remaining groups. G7 showed the lowest total protein among the groups. Blood ALT and AST were markedly higher in G7 and lowered in G2 and G6 than in the remaining groups. The highest ALT and AST levels were in tilapia grown in 15 ppt and continuously exposed to TAN (G7). Creatinine was increased in G1, G3, G7, and G8 and decreased in G2, G4, and G6 groups meaningfully. The urea levels were higher in G3, G5, and G7 than in G2, G4, G6, and G8 without significant differences with G1. The glucose level was significantly increased in G7 and decreased in G2, G4, G6, and G8. Further, groups G1, G5, and G7 had the highest cortisol levels, and the lowest levels were seen in the G2 group.

**Table 4:** Blood biomarkers of Nile tilapia reared in water salinity and ammonia exposure

Item	G1	G2	G3	G4	G5	G6	G7	G8	Two-way ANOVA (*P*-value)		
									Salinity	Ammonia	Interaction
Total protein (g/dl)	3.27 ± 0.04b	3.92 ± 0.04a	2.93 ± 0.05b	3.77 ± 0.01a	3.28 ± 0.05ab	3.42 ± 0.23ab	2.54 ± 0.02d	2.72 ± 0.03c	0.001	0.001	0.001
Albumin (g/dl)	1.38 ± 0.01b	1.48 ± 0.01a	1.30 ± 0.01b	1.45 ± 0.03a	1.36 ± 0.02b	1.38 ± 0.04b	1.24 ± 0.03c	1.30 ± 0.03c	0.001	0.001	0.001
Globulin (g/dl)	1.89 ± 0.03b	2.44 ± 0.03a	1.63 ± 0.04b	2.33 ± 0.03a	1.92 ± 0.03b	2.04 ± 0.18b	1.30 ± 0.03c	1.42 ± 0.01c	0.001	0.001	0.001
ALT (U/l)	39.96 ± 0.05b	32.37 ± 1.95c	37.76 ± 3.50b	36.80 ± 0.64b	41.40 ± 0.92b	33.22 ± 1.88c	44.46 ± 1.60a	38.88 ± 0.52b	0.001	0.001	0.001
AST (U/l)	31.57 ± 0.63b	21.23 ± 0.10c	30.31 ± 0.70b	31.79 ± 0.23b	30.85 ± 0.21b	21.38 ± 0.40c	38.40 ± 1.67a	32.97 ± 0.05b	0.001	0.001	0.001
Creatinine (mg/dl)	0.36 ± 0.02a	0.28 ± 0.02c	0.35 ± 0.02a	0.26 ± 0.02c	0.32 ± 0.02b	0.23 ± 2.02c	0.37 ± 0.02a	0.35 ± 0.02a	0.001	0.001	0.001
Urea (mg/dl)	3.28 ± 0.04ab	3.07 ± 0.04b	3.32 ± 0.04a	3.07 ± 0.04b	3.39 ± 0.04a	3.08 ± 2.04b	3.42 ± 0.04a	3.17 ± 0.04b	0.001	0.001	0.001
Glucose (mg/dl)	13.03 ± 0.34b	10.34 ± 0.10c	13.26 ± 0.61b	10.07 ± 0.03c	13.97 ± 0.23b	10.09 ± 0.12c	15.15 ± 0.60a	9.88 ± 0.22c	0.001	0.001	0.001
Cortisol (ng/ml)	45.23 ± 2.44a	38.77 ± 0.82c	43.28 ± 0.58ab	40.16 ± 0.17b	44.66 ± 0.27a	40.01 ± 0.08b	46.61 ± 0.82a	42.40 ± 0.72ab	0.001	0.001	0.001

Means ± S.E. in the same column with different letters differs significantly (*P* < 0.05). *ALT*, alanine aminotransferase; *AST*, aspartate aminotransferase; G1: 0 ppt/continuous total ammonia nitrogen exposure (TAN); G2: 0 ppt/intermittent TAN; G3: 5 ppt/continuous TAN; G4: 5 ppt/intermittent TAN; G5: 10 ppt/continuous TAN; G6: 10 ppt/intermittent TAN; G7: 15 ppt/continuous TAN; G8: 15 ppt/intermittent TAN

### Blood immunity

The lysozyme and phagocytic activities were significantly affected by water salinity, TAN, and their interaction (Table 5). The phagocytic activity and index were markedly higher in G2, G4, and G6 than in the remaining groups. Fish in G7 had lower phagocytic activity and index than in the G1, G3, G5, and G8 groups. The lysozyme activity was higher in G2, G4, G6, and G8 than in the G1, G3, G5, and G7 groups.

**Table 5:** Blood immunity of Nile tilapia reared in water salinity and ammonia exposure

	G1	G2	G3	G4	G5	G6	G7	G8	Two-way ANOVA (*P*-value)		
									Salinity	Ammonia	Interaction
Phagocytic activity (%)	3.22 ± 0.01b	3.37 ± 0.01a	3.08 ± 0.02b	3.44 ± 0.03a	3.24 ± 0.01b	3.61 ± 0.02a	2.90 ± 0.03c	3.20 ± 0.02b	0.001	0.001	0.001
Phagocytic index	1.31 ± 0.00b	1.50 ± 0.00a	1.19 ± 0.00b	1.40 ± 0.00a	1.28 ± 0.00b	1.36 ± 0.00a	1.00 ± 0.00c	1.28 ± 0.00b	0.001	0.001	0.001
Lysozyme activity (u/ml)	21.46 ± 0.17b	23.35 ± 0.68a	21.09 ± 0.08b	23.41 ± 0.59a	21.41 ± 0.15b	23.15 ± 0.54a	21.17 ± 0.07b	22.95 ± 0.34a	0.001	0.001	0.001

Means ± S.E. in the same column with different letters differs significantly (*P* < 0.05). *NBT*, nitro blue tetrazolium; G1: 0 ppt/continuous total ammonia nitrogen exposure (TAN); G2: 0 ppt/intermittent TAN; G3: 5 ppt/continuous TAN; G4: 5 ppt/intermittent TAN; G5: 10 ppt/continuous TAN; G6: 10 ppt/intermittent TAN; G7: 15 ppt/continuous TAN; G8: 15 ppt/intermittent TAN

## Discussion

Nile tilapia can grow in brackish water; however, high ammonia levels can impair health status and productivity (Dawood et al. 2021; Durigon et al. 2020). Under farming conditions, fish is expected to be stressed with continuous and intermittent ammonia exposure (Benli et al. 2008). The stress induced by ammonia exposure is markedly influenced by the accumulation level and duration of exposure (Esam et al. 2022). Besides, the water temperature and pH are also involved in forming toxic ammonia (Khoo et al. 1977; Kır et al. 2019; Sinha et al. 2015). Continuous and intermittent ammonia exposure is expected to affect the health status of Nile tilapia reared in different salinity levels. The increased osmoregulation energy cost and oxidative stress induced by high salinity and ammonia exposure cause metabolic and physiological alterations in fish’s entire body (Copatti and Baldisserotto 2021). Consequently, fish reveal interrupted responses such as antioxidative and immunity, which may result in high mortality rates (Abdo et al. 2022; Zhao et al. 2020). This study tested the growth performance, survival rate, antioxidative, blood bio-indicators, and immune responses in Nile tilapia reared in different salinity levels and continuous or intermittent ammonia exposure.

The high salinity combined with continuous ammonia exposure negatively affected the growth performance and survivability of Nile tilapia under the current trial circumstances. These results agree with Dawood et al. (2022b) and El-Leithy et al. (2019), who revealed reduced growth performance in Nile tilapia reared in long-term high salinity. Besides, Sánchez Ortiz et al. (2022) and Elbialy et al. (2021) stated similar reductions in the growth performances of Nile tilapia exposed to high ammonia. The consumed feed energy during osmoregulation caused by high salinity results in deteriorated metabolic and physiological responses, hence the growth performance (Chourasia et al. 2018; Herrera et al. 2009). Furthermore, ammonia exposure for a long time disrupts the physiological status of fish by inducing inflammation in the gills, intestines, and livers of fish (Evans et al. 2006). In this regard, the study tested the histological features of gills, intestines, and livers as well as their antioxidative capacity since stressed conditions induce oxidative stress coincided with high production of reactive oxygen species (ROS) (Zarantoniello et al. 2021), thereby, lipid peroxidation and inflammatory features (Zhou et al. 2020). Under high salinity and continuous ammonia exposure, inflammation in the intestines leads to inadequate digestion and absorption capacity (Cao et al. 2021; Qi et al. 2017). The study also indicated a high mortality rate in Nile tilapia reared in high salinity and exposed to ammonia. The low survival rate can be related to the fish’s failure of resistance to the stress induced by oxidative stress caused by high salinity and ammonia exposure.

High salinity and ammonia exposure are the main inducers of ROS involved in oxidative stress and lipid peroxidation of vital cellular components such as DNA (Shang et al. 2023; Zarantoniello et al. 2021). Accordingly, fish exhibit impaired cellular function accompanied by inflammation and irregular responses (Yan et al. 2021). The lipid peroxidation is expressed by the malondialdehyde formation (MDA) level (Marnett 1999), while the antioxidation capacity can be regulated by activating SOD, CAT, and GPX (Abogadallah 2010). In this study, the gills, intestines, and livers’ antioxidative capacity were impaired by high salinity and ammonia exposure. Consequently, under the present trial conditions, impaired respiration, osmoregulation, digestion, immunity, and antioxidative capacity were seen in Nile tilapia. In this regard, Tran-Ngoc et al. (2017) and Dawood et al. (2021) also illustrated that Nile tilapia exposed to high salinity showed interrupted histological features in the intestines, livers, and gills. Further, Nile tilapia (Benli et al. 2008), grass carp (*Ctenopharyngodon idella*) (Cao et al. 2021), and Japanese flounder (*Paralichthys olivaceus*) (Dong et al. 2013) exposed to high ammonia revealed abnormal histological features in the intestines, livers, and gills. The results indicated high MDA levels with low SOD, CAT, and GPX in the homogenates of gills, intestines, and livers of Nile tilapia reared in high salinity and exposed to continuous salinity. Similarly, increased MDA was observed in Nile tilapia reared in high salinity (Dawood et al. 2021) or ammonia exposure (Abdo et al. 2022). The variation in antioxidative responses among the different organs suggests that different organs may have different sensitivities to salinity and TAN exposure.

The intestines of fish are involved in the digestion and absorption of feed and are responsible for homeostasis, osmoregulation, and immune response (Filipp et al. 2019; Gupta et al. 2017). In heat-stressed fish, the intestinal mucosa aggregates extensive areas from animals’ bodies to relieve the impacts of heat stress (Cao et al. 2021; Tan et al. 2019). This study showed that the co-exposure to ammonia with high salinity revealed severe degeneration with catarrhal exudate in most structures of the intestine with hyperplasia in the tunica muscularis and inflammatory cell infiltration. Similarly, intestinal impairment was also observed in Nile tilapia (Li et al. 2021) exposed to ammonia. The inflammation induced by oxidative stress is probably the main reason for intestinal dysfunction in Nile tilapia exposed to high salinity.

Blood biochemical indices are reliable indicators that help assess the physiological condition and health status of animals suffering from biotic and abiotic stressors (Shahjahan et al. 2022). Continuous ammonia exposure and high salinity (15 ppt) are inducers for hepato-renal dysfunction and metabolic alterations, which can be predicted by measuring ALT and AST. The blood total protein, albumin, and globulin levels were markedly reduced in fish exposed to ammonia, high salinity (15 ppt), and both of them. Under high salinity conditions, common carp (Dawood et al. 2022a) showed reduced blood proteins. The oxidative stress induced by ammonia and high salinity results in loss of appetite malnutrition, thereby reducing protein metabolism in the fish’s entire body (Zarantoniello et al. 2021). Further, the study showed high levels of ALT and AST in Nile tilapia exposed to ammonia and high salinity continuously. These results are in line with Mangang and Pandey (2021), who indicated that *Osteobrama belangeri* exposed to ammonia displayed high ALT and AST levels. While Dawood et al. (2022a) reported high levels of ALT and AST in common carp exposed to high salinity. The high levels of ALT and AST indicate liver damage and the high release of these enzymes from the hepatocytes (Thapa and Walia 2007). Indeed, the toxic impacts of ammonia increase under high salinity conditions and result in high toxicity and liver dysfunction (Xu et al. 2021). The liver tissue detoxifies toxicants and ammonia, but severe toxicity induces inflammation and loss of liver function (Apte and Krishnamurthy 2011). In this regard, we detected the histological features of the liver after exposure to ammonia and high salinity in Nile tilapia. The co-exposure to ammonia with high salinity caused degenerative changes, pyknosis of hepatocytes nuclei, and congestion of blood vessels. These features may explain the increased levels of ALT and AST in the blood. The results are in line with Esam et al. (2022), who reported impaired liver histological features due to ammonia toxicity.

Disorders in kidney function can be detected by increased urea and creatinine levels in the blood (Toffaletti and McDonnell 2008). Increased creatinine level is related to the high energy metabolism in fish muscles (Bernet et al. 2001). The results showed high urea and creatinine levels in the blood of Nile tilapia exposed to ammonia and high salinity. Concurrent with this study, Esmaeili et al. (2017) reported that rainbow trout (*Oncorhynchus mykiss*) exposed to ammonia showed high creatinine and urea levels. Also, Waheed et al. (2020) reported that Nile tilapia reared in high salinity showed high urea and creatinine levels. The obtained results are probably related to the damage in the kidney tubules’ absorption capacity induced by inflammation resulting from oxidative stress (Abdeen et al. 2019). Concurrently, high protein loss from the kidney in the urine can explain the lowered total protein and albumin in fish exposed to ammonia and high salinity.

Cortisol is a primary, while glucose is a secondary stress response in fish bodies (Babitha and Peter 2010). Biotic and abiotic stressors, including ammonia toxicity and high salinity, are involved in glycolysis and gluconeogenesis induced by the cortisol effect, which increases due to the uprising of the hypothalamus (Eissa and Wang 2013; Simontacchi et al. 2009), leading to high glucose release in the blood as a source of energy (Ellis et al. 2012). The study showed increased glucose and cortisol levels in the blood of Nile tilapia exposed to ammonia, high salinity, and both. In line with the current study, Esmaeili et al. (2017) reported high glucose and cortisol levels in rainbow trout exposed to ammonia. While Zarantoniello et al. (2021) also stated high cortisol levels in Seabream (*Sparus aurata*) stressed with high salinity.

The fish’s immune system can be affected by biotic and abiotic stressors, including high salinity and ammonia exposure (Xu et al. 2021). The oxidative stress also extends to the damage of immune cells and the loss of their role in protecting fish from invaders (Kim et al. 2021). The results showed impaired lysozyme and phagocytic activities in Nile tilapia raised in high salinity and exposed to ammonia for an extended period.

## Conclusion

In summary, the water salinity, TAN, and their interaction affected the growth performance and survival rate of Nile tilapia. The reduction of the growth performance can be related to the impaired antioxidative capacity and immune response of tilapia reared in high salinity and exposed to TAN. High oxidative stress induced by high salinity and TAN exposure led to inflammatory attributes in the histological features of the gills, intestines, and livers of tilapia. Inappropriate blood bio-indicators (total, albumin, globulin, ALT, AST, creatinine, urea, glucose, and cortisol levels) were also seen in 15 ppt with continuous TAN exposure. Hence, Nile tilapia showed the possibility of growth with normal health status in brackish water (5–10 ppt); however, continuous TAN exposure can impair the productivity of tilapia, especially with high salinity (15 ppt).

## Data Availability

The datasets are available from the corresponding author upon reasonable request.
